# Advances and Evolving Challenges in Spinal Deformity Surgery

**DOI:** 10.3390/jcm12196386

**Published:** 2023-10-06

**Authors:** Ruchit V. Patel, Alexander G. Yearley, Hannah Isaac, Eric J. Chalif, Joshua I. Chalif, Hasan A. Zaidi

**Affiliations:** 1 Department of Neurosurgery, Brigham and Women’s Hospital, Boston, MA 02115, USA; rpatel80@bwh.harvard.edu (R.V.P.); alexander_yearley@hms.harvard.edu (A.G.Y.); echalif@mgb.org (E.J.C.); jchalif@bwh.harvard.edu (J.I.C.); 2 Harvard Medical School, Boston, MA 02115, USA

**Keywords:** spinal deformity, surgical approaches, operative challenges, minimally invasive surgery

## Abstract

Background: Surgical intervention is a critical tool to address adult spinal deformity (ASD). Given the evolution of spinal surgical techniques, we sought to characterize developments in ASD correction and barriers impacting clinical outcomes. Methods: We conducted a literature review utilizing PubMed, Embase, Web of Science, and Google Scholar to examine advances in ASD surgical correction and ongoing challenges from patient and clinician perspectives. ASD procedures were examined across pre-, intra-, and post-operative phases. Results: Several factors influence the effectiveness of ASD correction. Standardized radiographic parameters and three-dimensional modeling have been used to guide operative planning. Complex minimally invasive procedures, targeted corrections, and staged procedures can tailor surgical approaches while minimizing operative time. Further, improvements in osteotomy technique, intraoperative navigation, and enhanced hardware have increased patient safety. However, challenges remain. Variability in patient selection and deformity undercorrection have resulted in heterogenous clinical responses. Surgical complications, including blood loss, infection, hardware failure, proximal junction kyphosis/failure, and pseudarthroses, pose barriers. Although minimally invasive approaches are being utilized more often, clinical validation is needed. Conclusions: The growing prevalence of ASD requires surgical solutions that can lead to sustained symptom resolution. Leveraging computational and imaging advances will be necessary as we seek to provide comprehensive treatment plans for patients.

## 1. Introduction

As the global population continues to age, with a demographic transformation toward older age strata, there has been a corresponding increase in pathology involving the spinal column [1]. A major disease category affecting this patient population is adult spinal deformity (ASD), with a prevalence of up to about 70% in elderly patients [2]. ASD encompasses a heterogenous group of abnormalities affecting the thoraco-lumbar spinal column, which include diseases with acute onset (post-traumatic spinal deformity), exacerbation of pre-existing conditions (progressive adolescent idiopathic scoliosis in adulthood), or progressive conditions secondary to degenerative changes (de novo scoliosis and focal/global deformity from multi-level disc disease) [3,4,5]. The economic burden and aggregate disability from spinal deformities have led to the development of a spectrum of medical and surgical interventions to improve symptoms and quality of life [6,7].

Various non-operative methods are first-line treatments for ASD, including physical therapy, aerobic exercise, pharmacotherapy (non-steroidal anti-inflammatories, selective serotonin reuptake inhibitors, and anticonvulsants), and injections (steroid, trigger-point, and nerve root block). Procedural interventions available for ASD have evolved over the past few decades and are utilized when patients experience intractable pain, significant physical disability, neurological symptoms, or progression of abnormal spinal curvature with unsuccessful non-operative management [4,8]. Surgical options are broadly categorized into six subtypes which attempt to achieve correction based on the extent of deformity: decompression, decompression and posterior fusion with limited instrumentation, decompression and posterior fusion with lumbar curve instrumentation, decompression and anterior/posterior fusion, fusion with thoracic instrumentation, and osteotomies [8]. Management algorithms have combined spinal characteristics (e.g., fixed versus flexible spine) with radiographic parameters, such as pelvic tilt, to guide the choice of an appropriate surgical option. Across these approaches, surgical planning, instrumentation, and techniques have evolved, incorporating advances such as minimally invasive surgery (MIS) to help reduce morbidity associated with open spinal correction [9]. However, there remain challenges in both open and minimally invasive techniques, such as appropriate patient identification, selection of surgical approaches, post-operative complications, and limitations to the degree of achievable correction [10,11].

**Figure 1:**
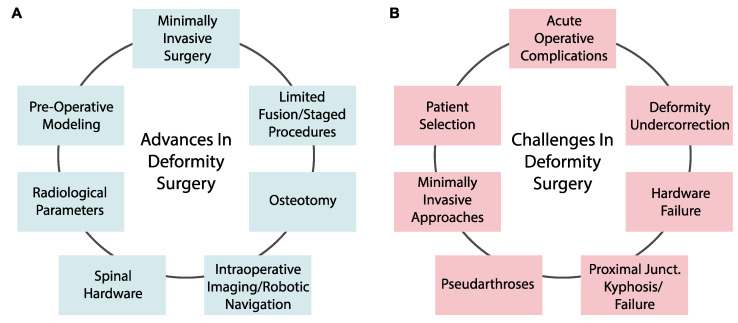
(**A**) Advances in surgical approaches for adult spinal deformity correction. (**B**) Ongoing barriers and challenges impacting adult spinal deformity procedures and clinical outcomes.

Given the critical role of surgical options for ASD, we sought to characterize advances in surgical techniques for deformity correction and targets for improvement. Through a comprehensive review across four databases (PubMed, Embase, Web of Science, and Google Scholar), we focus on developments in pre-operative planning, open surgical procedures, and minimally invasive protocols (Figure 1A). Further, we review contributors to morbidity after deformity correction and ongoing challenges with integrating new spinal approaches (Figure 1B). 

A critical lens toward ASD surgery will help ensure continued development of safer, inclusive, and more effective treatment options.

## 2. Advances in Surgical Approaches

### 2.1. Radiological Parameters and Scoring Systems

**Figure 2:**
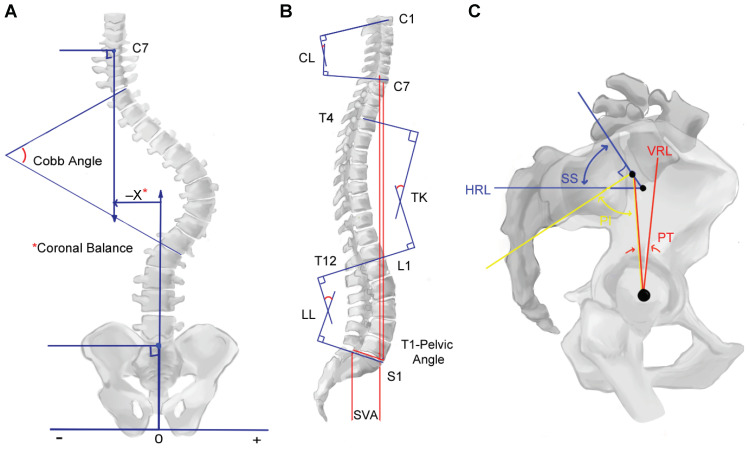
Radiographic metrics used to determine severity of spinal deformity and target degree of correction in (**A**) coronal, (**B**) sagittal, and (**C**) pelvic views. CL: cervical lordosis, HRL: horizontal reference line, LL: lumbar lordosis, PI: pelvic incidence, PT: pelvic tilt, SS: sacral slope, SVA: sagittal vertical axis, TK: thoracic kyphosis, VRL: vertical reference line. * Coronal balance. Adapted from Smith et al., *Journal of Neurosurgery* (2019). All illustrations performed by author HI.

The development of quantifiable pre-operative parameters from plain film and computed tomography (CT) imaging has enabled patient risk stratification and identification of targets for deformity correction. Using whole spine posterior–anterior and lateral views, several parameters can be calculated: regional metrics (Cobb’s angles for cervical lordosis (CL), thoracic kyphosis (TK), thoracolumbar kyphosis, and lumbar lordosis (LL)), sagittal spinopelvic metrics (sagittal vertical axis (SVA), pelvic incidence (PI), pelvic tilt (PT), sacral slope (SS), and T1 pelvic angle), and coronal metrics (Cobb’s angles for scoliotic curves and coronal balance) [12,13] (Figure 2). 

**Figure 3:**
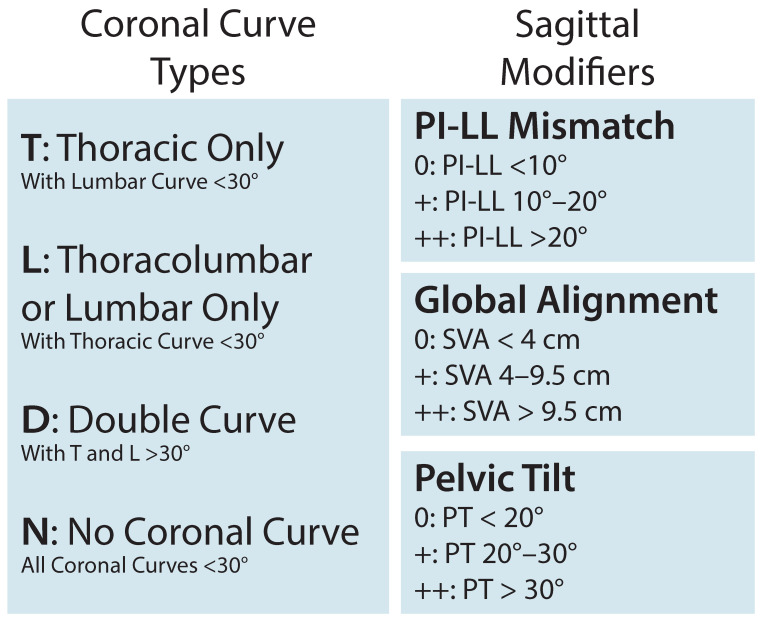
Scoliosis Research Society-Schwab classification scheme to guide treatment decisions for adult spinal deformity. LL: lumbar lordosis, PI: pelvic incidence, PT: pelvic tilt, SVA: sagittal vertical axis.

Several of these parameters have been integrated, such as mismatch between PI and LL, to create the validated Scoliosis Research Society (SRS)-Schwab classification scheme to guide treatment decisions (Figure 3). 

This system incorporates both coronal and sagittal deformity modifiers, focusing on spinopelvic metrics to capture alignment. SRS-Schwab classification has demonstrated robust reliability across institutions, with a PI-LL mismatch of <10°, SVA < 4 cm, and PT < 20° predictive of positive clinical outcomes, reduced disability, and improved health-related quality of life scores [14,15,16]. Subsequent iterations with new scoring systems like the Global Alignment and Proportion (GAP) score have sought to better predict post-operative mechanical complications and risk of revision surgery, which are aspects that the SRS-Schwab system does not address [17]. Radiographic targets continue to be optimized as we better understand how comorbidities and demographic variables such as age modify classification systems. For example, previous analysis of post-operative disability across age and SRS-Schwab strata demonstrated age-adjusted spinopelvic parameters, with younger patients requiring more aggressive deformity correction for symptom improvement [18,19]. GAP score adjustment using body mass index and bone density has further shown better predictive performance compared to unadjusted GAP scores [20]. While additional scoring systems continue to be explored, quantitative classification has enabled greater identification and standardization of surgical approaches that account for the mechanics underlying ASD, targets for alignment, and technique selection.

### 2.2. Pre-Operative Modeling

**Figure 4:**
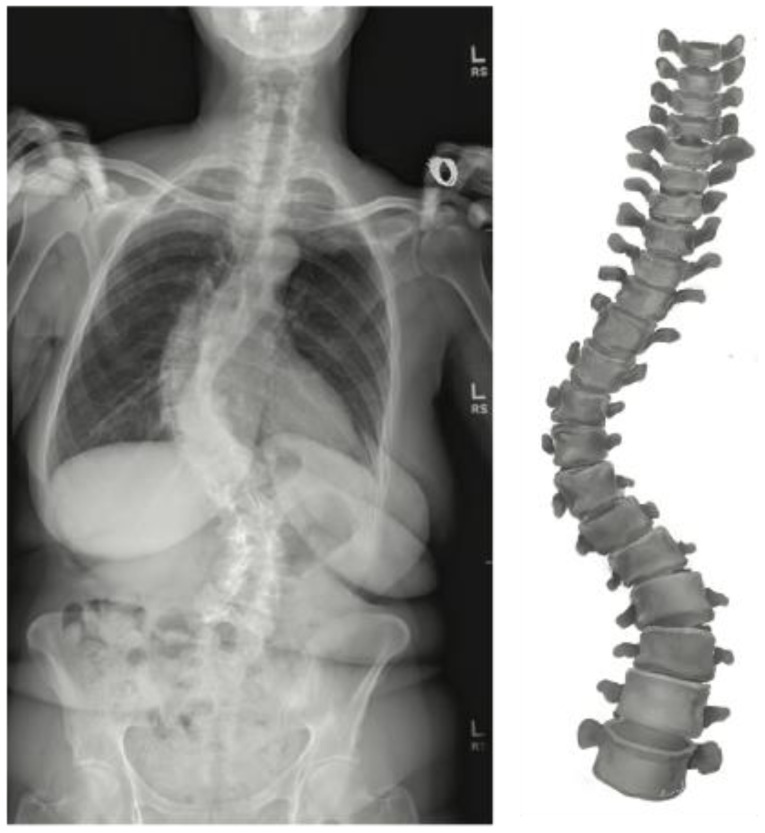
Generation of three-dimensional spinal models for pre-operative planning. Radiographic views can be integrated to create a virtual model that can be examined and manipulated.

The accessibility of computational workflows has led to the development of pre-operative tools that can better predict intra-operative challenges and post-operative complications. Three-dimensional reconstruction, both in virtual and physical space, has facilitated pre-operative visualization (Figure 4).

CT reconstruction captures bony changes such as facet defects or osteophyte complexes with high fidelity, while enabling precise calculations on pedicle diameter and spinal angles [21]. To capture spine anatomy in upright weight-bearing positions, low-dose stereoradiography has also been introduced. This technology generates three-dimensional reconstructions of bony structures from two-dimensional images while minimizing radiation [22]. Stereoradiography has shown high accuracy in spinal measurements versus CT reconstruction, especially in evaluating scoliotic curvature and sagittal parameters [23,24,25,26]. Although proof of concept has been established, the clinical utility and impact of stereoradiography continues to be investigated. Physical spine models printed from virtual three-dimensional reconstructions have also been shown to improve operative planning, especially in cases with complex or anomalous anatomy [27]. Three-dimensional printing has further enabled the creation of patient-specific hardware guides, thus im proving screw placement accuracy and reducing surgical time [28,29].

Large clinical ASD patient datasets have further generated predictive models built on machine and deep learning architectures. One example where these computational methods have been applied is to evaluate the risk of perioperative blood transfusions following ASD surgery [30,31]. Post hoc analysis has identified salient variables associated with increased transfusion risk, including operative time, hematocrit, and patient weight. Machine learning models have also been developed to attempt prediction of major complications like proximal junctional kyphosis (PJK), proximal junctional failure (PJF), and pseudarthroses [32,33,34]. Certain important features have emerged, including patient age, lower/upper vertebral locations, implant types utilized, and pre-operative radiological parameters. Predicting the risk of complications before entering the operating room can improve pre-operative counseling while helping to minimize morbidity associated with deformity correction.

### 2.3. Minimally Invasive Surgery

**Figure 5:**
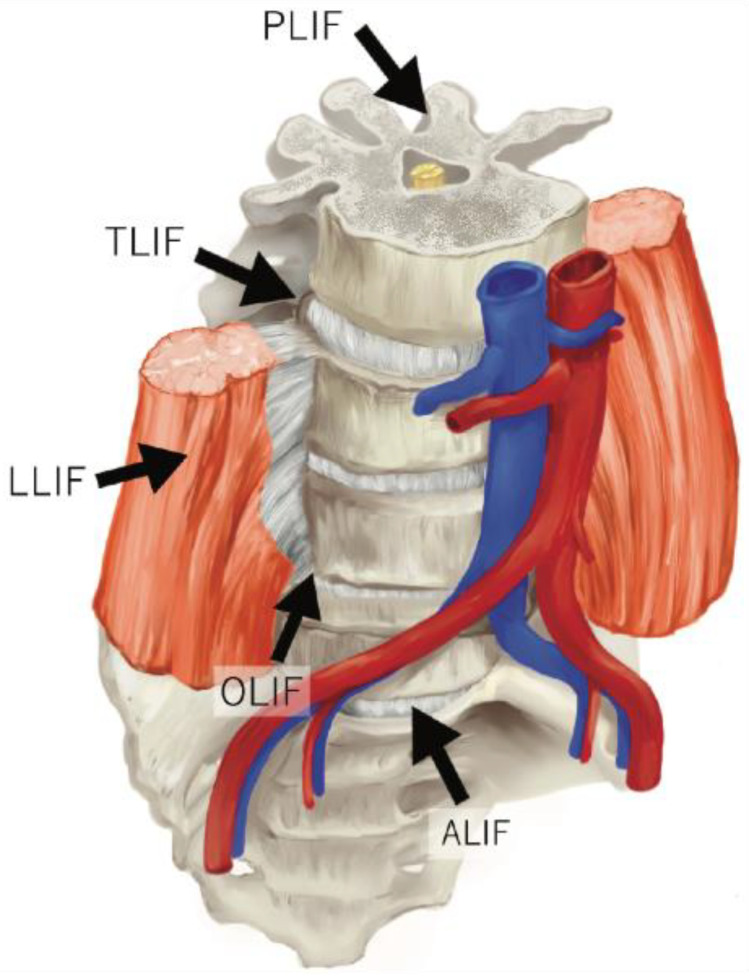
Application of minimally invasive techniques to lumbar spinal approaches. ALIF: anterior lumbar interbody fusion, LLIF: lateral lumbar interbody fusion, OLIF: oblique lumbar interbody fusion, PLIF: posterior lumbar interbody fusion, TLIF: transforaminal lumbar interbody fusion. Adapted from Mobbs et al., *Journal of Spine Surgery* (2015). All illustrations performed by author HI.

Surgical techniques for ASD correction have also advanced, with MIS approaches that leverage endoscopic tools being utilized with greater frequency. MIS aims to preserve anatomy, reduce morbidity, and accelerate patient return to function with smaller incisions, all features of great interest for deformity correction [35,36]. MIS has been applied to several lumbar spinal approaches, such as lateral lumbar interbody fusion (LLIF), oblique lumbar interbody fusion (OLIF), transforaminal lumbar interbody fusion (TLIF), posterior lumbar interbody fusion (PLIF), and anterior lumbar interbody fusion (ALIF) (Figure 5), as well as techniques such as anterior longitudinal ligament (ALL) release, percutaneous screws, and prone/lateral positioning [37,38,39,40]. 

To improve correction of sagittal and spinopelvic parameters in ASD, circumferential MIS (cMIS) was developed, which combines anterior column support (e.g., LLIF/OLIF) with percutaneous posterior instrumentation [41,42]. While radiographic correction data from cMIS approaches remain heterogenous, cMIS appears to correct sagittal imbalance even in cases of severe ASD [43,44]. Clinical outcomes are similarly variable, with studies reporting unchanged to improved patient outcomes following cMIS versus open procedures. In several studies, at one year post-operatively, patients who received cMIS had similar disability scores and spinopelvic parameters compared to patients where open correction was performed, with no difference in complication rates [45,46,47]. However, in one study with longer follow-up intervals, patients who received cMIS had a significantly lower incidence of major complications like PJK/PJF, pseudarthroses, and hardware failure [48]. Given the variability in documented radiographic and clinical correction with MIS techniques, patient selection algorithms, like the minimally invasive spine deformity surgery 2 (MISDEF2) protocol, have been developed to identify patients who can benefit from an MIS approach [49]. MISDEF2 combines deformity characteristics (fixed versus flexible) and radiographic parameters (PI-LL mismatch, SVA, PT, and coronal Cobb angle) to place patients into four classes to guide the selection of spinal implants and MIS versus open techniques. 

### 2.4. Limited Fusions and Staged Procedures

Evolving surgical instrumentation and techniques have also enabled more targeted surgical correction, especially in cases of mild to moderate spinal deformity. In patients with small spinal radiographic changes and good sagittal balance, short fusions limited to the region of deformity showed fewer perioperative complications like hardware failure [50,51]. Applying a minimum necessary approach to deformity instrumentation can also be seen in the development of staged correction procedures [52,53,54,55]. These approaches generally utilize two stages: first, anterior column support is performed, after which the patient’s symptoms and degree of spinal correction are assessed before deciding whether to proceed with posterior instrumentation. Staged procedures minimize time under anesthesia for each individual procedure while offering a dynamic assessment of symptom improvement as correction is taking place [55]. While one study employing a staged approach demonstrated significantly lower rates of hardware failure compared to un-staged open deformity correction, another study showed no significant difference in staged versus un-staged cMIS procedures [56,57]. Additional work is needed to characterize the clinical benefit in specific patient populations while identifying potential logistical challenges in implementing such procedures.

### 2.5. Osteotomy

In cases of severe ASD, osteotomies are an important method for correction. While the first osteotomies were performed many decades ago, improvements in intra-operative tools and techniques have advanced their therapeutic benefit. The extent of an osteotomy is defined by six anatomic grades ranging from least aggressive (grade 1) to most aggressive (grade 6) based on the structures being removed (Table 1) [58].

Although a higher-grade osteotomy involving complete vertebrae and discs may be required for extensive deformity correction, larger resections increasingly destabilize the spine and risk significant post-operative complications. Three major types of osteotomies are performed: posterior column (Smith-Peterson osteotomy [SPO] and Ponte osteotomy), pedicle subtraction osteotomy (PSO), and vertebral column resection (VSR) [59,60,61,62]. Posterior column osteotomies involve the facet joints, lamina, and posterior ligaments, while PSO and VSR are considered three-column osteotomies that involve bone resection, making these more technically challenging. Recently, PSO and VSR have been declining in use due to their high complication rate and significant morbidity [63,64,65]. Despite this, for selected cases when extensive correction is required, advances in intra-operative navigation have improved the safety of three-column osteotomies. Three-dimensional pre-operative planning or intra-operative navigation using CT-based methods or O-Arm guidance can enhance accuracy in bone cuts, while providing real-time anatomic feedback of surrounding structures to reduce complications [66,67,68,69]. Optimizing navigation during osteotomies is, therefore, an important step toward improving safety for patients when surgical options are limited.

### 2.6. Intraoperative Imaging and Robotic Navigation

Tools for intraoperative visualization and surgical navigation have been integrated across a range of ASD correction procedures. Spinal endoscopy and video visualization are instrumental for MIS approaches, enabling high-fidelity deep tissue views with smaller incisions [70]. Randomized trials have been performed on the use of spinal endoscopy for discectomy, demonstrating non-inferiority to open alternatives with benefits in reducing intraoperative blood loss, hospital length of stay, and time to recovery [71,72,73]. Similar to the impetus for spinal endoscopy, miniature camera systems have been developed, which clip onto tubular retractors to allow improved operative visualization. Three-dimensional imaging techniques have moved to the intra-operative space, such as CT-guided and augmented reality-based placement of pedicle screws. For example, real-time three-dimensional evaluation has helped identify mispositioned screws, lowering the rate of revision surgery [74,75,76,77]. Robotic navigation has built on these advances, improving pedicle screw placement accuracy compared to conventional approaches while reducing image-related radiation exposure [78,79,80]. Further, robotic guidance has been helpful in planning cases with difficult pedicle anatomy or if prior instrumentation has been performed. Nevertheless, the impact of these navigation methods on surgical performance and clinical outcomes specifically for ASD correction remains to be seen through validated trials.

### 2.7. Hardware

**Figure 6:**
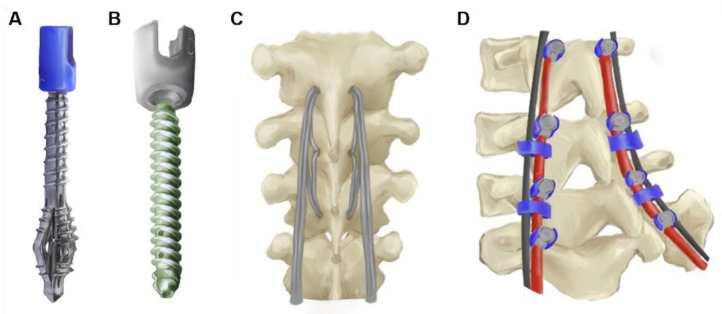
Novel hardware options developed to improve ASD correction. These include (**A**) expandable and (**B**) fenestrated cement-injectable screws, (**C**) junctional tethers at the uppermost instrumented vertebrae, and (**D**) multi-rod configurations.

Advances in devices and hardware available for ASD correction can make these procedures more accessible to a broader group of patients. Osteopenia and osteoporosis, which tend to co-occur in patients with ASD, pose significant limitations in the successful placement of anterior column hardware and pedicle screws [81]. Low bone density has been associated with poor stability of implants, difficulty with implant fixation, and post-operative hardware failure with an increased risk of revision surgery [82,83,84]. New pedicle screw designs such as expandable, fenestrated cement-injectable, and conical screws, as well as screw anchors, have the potential to improve fixation in osteoporotic bone [85,86,87,88] (Figure 6A,B). In cases when longer spine instrumentation is required, new screws for sacropelvic fixation have been developed to address distal screw failure and pseudarthrosis. For example, S2-alar-iliac screws reduce soft tissue dissection, can more easily connect to posterior instrumentation, and increase mechanical strength while reducing the rate of reoperation [89,90]. Junctional tethers are another technique which continues to be explored to reduce the risk of PJK/PJF [91,92] (Figure 6C). Previous studies found that weaving tethers to dissipate stress on adjacent spinal segments was safe and showed early signs of lowering long-term PJK risk [93,94]. Finally, posterior rod augmentation with multi-rod configurations has been studied to reduce the risk of rod fracture by providing additional structural support (Figure 6D). 

In small cohorts, multi-rod techniques showed lower rates of rod fracture and pseudarthrosis compared to standard two-rod techniques [95,96,97]. Device innovation will play an integral role in tailoring deformity correction procedures for individual patient presentations.

## 3. Challenges in Spinal Deformity Surgery

### 3.1. Patient Selection

Changing patient demographics and comorbidity rates, especially amongst those with ASD, have increasingly made it challenging to identify appropriate surgical candidates [98]. With the mean age of de novo scoliosis at 70.5 years and degenerative hyperkyphosis at 78.3 years, there are few validated metrics that predict which patients can tolerate and benefit from a significant surgical correction [5]. However, it remains true that patients with more severe ASD or associated disability benefit the most from surgical correction [99]. One proposed model combined patient demographics with features of the surgical approach to predict the risk of intraoperative bleeding and surgical duration [100]. Another study developed an ASD frailty index, which showed increased risk of major complications and hospital length of stay in more frail patients [101]. Additional work is needed to better understand how to risk stratify clinically complex or frail patients while also understanding how pre-operative health optimization can influence clinical outcomes.

### 3.2. Acute Operative Complications

ASD corrections are often large, surgically complex procedures that involve multiple entry points, spinal manipulation, and instrumentation. As a result, these procedures carry a high risk of acute intra- and post-operative complications, such as cardiac (myocardial infarction and cardiac arrest), pulmonary (pneumonia and pulmonary embolism), vascular (deep vein thromboses), neurologic (cauda equina and nerve root injury), and infectious (sepsis, superficial infection, and thrombophlebitis) etiologies [102,103]. Surgical site infections are one category that can result in significant morbidity and mortality. For ASD procedures, surgical site infections have been reported in up to 10% of cases, with risk factors for infection including prior history of a site infection and elevated body mass index [104,105,106,107]. A systems-based approach that incorporates patient comorbidity optimization, timely administration of antibiotics, surgical sterile technique, and post-operative wound care has been shown to reduce the incidence of surgical site infections [108]. 

Blood loss associated with ASD surgery is another source of complications. Increased exposure to blood products and large fluid shifts can lead to transfusion-associated reactions and severe coagulopathies that can result in multi-organ sequelae [109]. Several strategies have been investigated to mitigate blood loss during ASD surgery. Intra-operative use of antifibrinolytics like tranexamic acid has been shown to reduce the need and volumes of post-operative transfusions [110,111,112]. Patient positioning has also been explored to decrease bleeding risk by reducing intra-abdominal pressure, but efficacy has been variable. Positions include Trendelenburg for lower thoracic/lumbar spine procedures, reverse-Trendelenburg for cervical spine procedures, Jackson table/Wilson frame with free suspension of the abdomen, and jackknife positions [113,114,115]. Other areas that have been explored to minimize blood loss include bipolar electrocautery and topical hemostatic agents like bone wax [116,117]. To monitor patient coagulation status more accurately, viscoelastic point-of-care methods are also being tested, with initial data demonstrating a reduction in total blood products administered [118,119].

Dual surgeon approaches, which are utilized in other surgical specialties, may also play an instrumental role in helping to reduce the incidence of operative complications [120]. The cognitively and physically demanding nature of ASD corrections may benefit from collaboration within the operating room. Compared to single-surgeon spinal cases, dual-surgeon cases have been shown to decrease blood loss and operative time [121,122,123,124]. Supporting operating room and staffing workflows that facilitate dual-surgeon cases in the setting of certain complex or high-risk ASD procedures may, therefore, prove to be a beneficial strategy.

### 3.3. Undercorrection

Although undercorrection of ASD occurs with a relatively low frequency, it is associated with poor health-related quality-of-life outcomes [125]. ASD undercorrection is often a result of targeting sagittal alignment parameters without accounting for patient age. Across sagittal parameters, such as PT, PI-LL mismatch, LL-TK mismatch, and SVA, younger patients were found to have lower radiographic thresholds at which significant disability and poor health-related quality of life were reported compared to older patients [19]. As a result, a more aggressive degree of deformity correction is likely required in younger populations to improve functional outcomes [125]. Individualized pre-operative planning and age-specific radiological assessment are, therefore, needed to account for age-related changes across the musculoskeletal system.

### 3.4. Hardware Failure

An integral component of ASD surgery is the instrumentation and hardware required to achieve deformity correction. The estimated rate of hardware failure is variable, ranging up to about 50% in some cohorts [126]. Several factors influence the likelihood of failure, including patient factors such as age, bone density, and comorbidities, as well as aspects of the procedure itself such as complexity of the correction and numbers of spinal levels fused [127,128,129]. Sources of hardware failure are variable, with rod fracture, painful/prominent implants, screw breakage, and screw loosening being some of the most frequently reported [103]. In particular, rod fractures have been subjected to intensive investigation, given the frequent need for revision surgery and significant deleterious consequences on patient recovery. This is especially seen when aggressive sagittal parameter correction is performed, as procedures like PSO are associated with the highest incidence of rod fractures [103]. Multi-rod configurations and customizable rods with greater angular bending may help ensure the utilized hardware remains stable across the lifespan [97,130].

### 3.5. Proximal Junctional Kyphosis and Failure

PJK and PJF, which refer to the development of abnormal kyphotic curvature at the uppermost instrumented vertebrae, are challenging complications of ASD surgery. The incidence of PJK/PJF varies and has been reported to be between 20 and 40% in most studies, depending on the patient population and extent of deformity correction [131]. A majority of patients with PJK have clinically significant signs within 18 months after surgery, though the time course can differ based on the rate of kyphosis progression or vertebral body structural failure [132]. While there is a lack of consensus regarding which patients with PJK should undergo surgery, patients with significant pain, neurological findings, or ongoing disability are considered for revision surgeries [133,134]. Several risk factors for PJK have been identified, including older age, large sagittal parameter abnormalities, low bone density, fusion to the sacrum/pelvis, and overcorrection of lumbar lordosis [131,134,135,136].

A consideration when evaluating PJK is selection of patient populations who can benefit from a revision operation. As radiographic findings may not completely correlate with patient symptoms, classification schemes have been developed to help establish consensus on treating PJK. The Boachie-Adjei system breaks PJK down into type of failure (e.g., ligamentous, bone, and implant–bone interface), degree of kyphosis, and presence of spondylolisthesis (Table 2) [137,138].

However, this system does not provide correlation with symptoms or guide treatment decisions. The Hart-International Spine Study Group scale built on this by grading PJK on six criteria: neurological deficits, pain, issues with instrumentation, degree of kyphosis, fracture/movement of upper instrumented vertebrae, and level of PJK involvement (Table 3) [139].

This scoring system has been validated against health-related quality of life scores, overall pain, and likelihood of revision surgery, establishing a threshold at which patients should be considered for revision [140,141].

Given the high incidence of PJK/PJF, several augmentation strategies for posterior instrumentation have been explored. Prophylactic teriparatide has been examined to improve bone mineral density in patients prior to deformity correction. In a prospective study, patients who received teriparatide had significantly increased bone mineral content with an almost 70% reduction in bone failure PJK compared to untreated patients at two years post-surgery [142]. Vertebral body cement augmentation is another tool that has been studied in both cadaveric models and patients. Studies showed that cement augmentation reduced junctional fractures in spinal models following long instrumentation and led to significant clinical reduction in incidence of PJK [143,144,145]. As previously mentioned, tethers, which are woven through the proximal junction of the deformity, have also been explored, with early data demonstrating low rates of symptomatic PJK and need for revision surgery [91,92,93,94]. The use of spinal hooks versus pedicle screws at upper instrumented sites has been explored, as more rigid anchor types have been associated with the development of PJK. Hooks were shown to significantly reduce the incidence of PJK compared to pedicle screws and were thought to better accommodate the dynamic post-operative forces placed on an instrumented spine [146,147,148,149]. Utilizing age-adjusted spinopelvic alignment goals further prevented overcorrection and stress placed on junctional sites [150]. Finally, modification of surgical techniques by preserving posterior tension bands while using fewer implants and more flexible materials may reduce the likelihood of PJK progression [131].

### 3.6. Pseudarthrosis

Pseudarthrosis refers to the failure of bone fusion after ASD correction and is another complication associated with significant cost and morbidity. Across ASD, the rate of pseudarthrosis is approximately 6% and accounts for a large portion of revision surgeries [151,152]. The risk factors for incomplete fusion are similar to those for PJK/PJF, including older age, underlying comorbidities, smoking, large thoracic kyphotic angles, and long construct lengths [151,153]. Even in situations when revision surgeries are performed, the rate of recurrent pseudarthrosis is high, affecting up to 50% of patients [154]. Various graft and biological materials have been under investigation to improve bone healing and drive hardware integration, but additional clinical data are needed to characterize their impact on long-term fusion [155]. Further, for high-risk patients, computational models that leverage patient and surgical variables might help guide pre-operative counseling and planning [34].

### 3.7. Minimally Invasive Approaches

While MIS approaches have expanded the options available for patients with ASD, there are limitations to performing minimally invasive techniques in those who require aggressive deformity correction. Initial applications of MIS for ASD showed challenges in the correction of sagittal parameters in patients, with open procedures achieving a greater degree of correction [36,156,157]. The development of more complex MIS approaches, like cMIS which can reach sagittal planes, have addressed some of these concerns, as previously mentioned. However, the efficacy of MIS or rate of conversion to open procedures in patients with certain severe deformities or large fusion masses remains to be seen. Further, MIS techniques carry new training requirements and start-up cost considerations [158]. As improvements in medical devices and hardware drive the growth in MIS approaches, clinical trial data comparing these approaches to open techniques will be critical to ensure appropriate procedure selection for patients with a particular ASD.

## 4. Future Directions

Surgical approaches for ASD have seen remarkable evolution driven by an expanding patient population and rapid operative advances. As innovation in health technology and data science becomes more accessible, these techniques can be leveraged to improve ASD correction outcomes. For pre-operative assessment, three-dimensional motion analysis paired with traditional radiographic parameters may offer more nuanced views on spinal mechanics and regions where correction is required [159]. These forms of imaging can further translate to precision hardware such as patient-specific rods that can lower the risk of rod fracture and hardware failure [160]. Advanced imaging also has several intra-operative applications, including real-time modeling and simulations through augmented reality to ensure safe and effective hardware placement [161,162]. Additionally, ASD patient identification and risk stratification can benefit from more powerful machine and deep learning architectures, which enable clustering that is independent of current descriptive categories. As computational classification schemes can more accurately reflect patient phenotype, these technologies can guide the development of management algorithms that incorporate patient health status and comorbidities, which can facilitate more tailored surgical approaches [163,164]. Across all of these domains, striking close partnerships between academics and industry sponsors, as well as clinicians delivering non-operative management, will be critical to improve the array of solutions available.

## 5. Conclusions

The growing global prevalence of ASD has necessitated effective surgical options that can address patient symptoms and deliver long-term correction. Several advances have fueled the growth of deformity surgery, such as standardized pre-operative evaluation metrics, three-dimensional spinal modeling, complex minimally invasive approaches, targeted and staged corrections, safer osteotomies, intraoperative navigation, and advanced hardware. For continued growth of ASD surgical options, several challenges will be important to address, including appropriate patient selection, ensuring adequate age-specific correction, improving minimally invasive approaches for severe deformities, and reducing significant operative complications such as infections, blood loss, hardware failure, PJK/PJF, and pseudarthroses. With the significant positive impact ASD surgery can have on reducing disability and improving quality of life, comprehensive outcome data, validated clinical trials, and continued innovation will be integral to advance these spinal interventions.

## Figures and Tables

**Table 1:** Classification system for osteotomies and corresponding anatomic regions removed.

	Anatomic Regions Resected
Grade 1	Partial facet joint (inferior facet and joint capsule)
Grade 2	Complete facet joint (superior and inferior facets with ligamentum flavum removal)
Grade 3	Pedicle and partial body (posterior vertebral body partial wedge resection and posterior vertebral elements)
Grade 4	Pedicle, partial body, and disc (posterior vertebral body wider wedge resection, posterior vertebral elements, and portion of >1 endplate and intervertebral disc)
Grade 5	Complete vertebra and both adjacent discs
Grade 6	Multiple vertebrae and discs

**Table 2:** The Boachie-Adjei classification system for proximal junctional kyphosis.

	Category	Description
Type	Type 1	Disc and ligamentous failure
	Type 2	Bone failure
	Type 3	Implant/bone failure
Grade	Grade A	Proximal junction increase of 10°–19°
	Grade B	Proximal junction increase of 20°–29°
	Grade C	Proximal junction increase ≥30°
Spondylo- listhesis	PJF-N	No spondylolisthesis present above the uppermost instrumented vertebra
	PJF-S	Spondylolisthesis present above the uppermost instrumented vertebra

**Table 3:** The Hart-International Spine Study Group scale for proximal junctional kyphosis. PLC: posterior ligamentous complex, UIV: uppermost instrumented vertebra, VAS: visual analog scale.

	Characteristic	Severity Score
Neurological Deficit	None	0
	Radicular Pain	2
	Myelopathy or Motor Deficit	4
Focal Pain	None	0
	VAS ≤ 4	1
	VAS ≥ 5	3
Instrumentation Problem	None	0
	Partial Fixation Loss	1
	Prominence	1
	Complete Fixation Loss	2
Change In Kyphosis	0–10°	0
	10°–20°	1
	>20°	2
	PLC Failure	2
UIV Changes	None	0
	Compression Fracture	1
	Burst/Chance Fracture	2
	Translation	3
Level of UV	Thoracolumbar Junction	0
	Upper Thoracic	1

## Data Availability

No new data were created or analyzed in this study. Data sharing is not applicable to this article.
